# Exo-ethylene application mitigates waterlogging stress in soybean (*Glycine max* L.)

**DOI:** 10.1186/s12870-018-1457-4

**Published:** 2018-10-22

**Authors:** Yoonha Kim, Chang-Woo Seo, Abdul Latif Khan, Bong-Gyu Mun, Raheem Shahzad, Jeung-Woo Ko, Byung-Wook Yun, Soon-Ki Park, In-Jung Lee

**Affiliations:** 1 Division of Plant Biosciences, Kyungpook National University, Daegu, 702-701 South Korea; 2 UoN Chair of Oman’s Medicinal Plants & Marine Natural Products, University of Nizwa, 616 Nizwa, Oman

**Keywords:** Antioxidant, Gibberellin, Photosynthesis efficiency, Plant growth regulator, Reactive nitrogen species, Reactive oxygen species

## Abstract

**Background:**

Waterlogging (WL) is a key factor hindering soybean crop productivity worldwide. Plants utilize various hormones to avoid various stress conditions, including WL stress; however, the physiological mechanisms are still not fully understood.

**Results:**

To identify physiological mechanisms during WL stress, different phytohormones, such as ethephon (ETP; donor source of ethylene), abscisic acid, gibberellins, indole-3-acetic acid, kinetin, jasmonic acid, and salicylic acid were exogenously applied to soybean plants. Through this experiment, we confirmed the beneficial effects of ETP treatment. Thus, we selected ETP as a candidate hormone to mitigate WL. Further mechanistic investigation of the role of ETP in waterlogging tolerance was carried out. Results showed that ETP application mitigated WL stress, significantly improved the photosynthesis pigment, and increased the contents of endogenous GA_s_ compared to those in untreated plants. The amino acid contents during WL stress were significantly activated by EPT treatments. The amino acid contents were significantly higher in the 100 μM ETP-treated soybean plants than in the control. ETP application induced adventitious root initiation, increased root surface area, and significantly increased the expressions of glutathione transferases and relative glutathione activity compared to those of non-ETP-treated plants. ETP-treated soybeans produced a higher up-regulation of protein content and glutathione S-transferase (GSTs) than did soybeans under the WL only treatment.

**Conclusions:**

In conclusion, the current results suggest that ETP application enabled various biochemical and transcriptional modulations. In particular, ETP application could stimulate the higher expression of GST3 and GST8. Thus, increased GST3 and GST8 induced 1) increased GSH activity, 2) decreased reactive oxygen species (ROS), 3) mitigation of cell damage in photosynthetic apparatus, and 4) improved phenotype consecutively.

**Electronic supplementary material:**

The online version of this article (10.1186/s12870-018-1457-4) contains supplementary material, which is available to authorized users.

## Background

The world’s climate has been rapidly changing due to increased carbon dioxide concentration in the atmosphere [1]. Such changes in global climate have severely influenced agricultural land and increased detrimental abiotic stresses such as drought, salinity, thermal, ultra violet, ozone, and flooding stresses. These environmental stresses hinder crop productivity [2–6]. Some of the underlying stress mechanisms are still not fully understood for all crops. Understanding stress tolerance and mitigation responses, and how to further improve these are important to ensure sustainable agriculture production for the ever-increasing human population [4–7]. Among abiotic stresses, flooding is caused by increased water levels in the cultivation field [8]. Flooding negatively influences the physiological functions of plants, which leads to reduced photosynthesis, imbalance in phytohormones, reduced nutrients uptake, premature fruit drop, stunted growth, and reduced yield [9, 10]. For acclimation to hypoxia, plants morphologically change their intercellular formation by developing aerenchyma cells in plant roots, which trigger several signal regulators such as nitric oxide, reactive oxygen species (ROS), and plant hormones especially ethylene (ET) [11]. Endogenous ET has been associated with the formation of aerenchyma cells however, this depends on the level, intensity, and duration of the flooding stress [12]. Both types of primary aerenchyma, e.g., schizogenous and lysigenous formations, are promoted in subaquatic rice plants, maize, barley, and wheat roots under waterlogged conditions. In contrast, in dryland crop plants such as soybean, the secondary type of aerenchyma is found and differentiated from the secondary meristem, and is observed in the stem, taproot, hypocotyl, adventitious roots, and root nodules [13–16]. In addition, this further signals a plethora of physiological networks associated with plant growth and flooding stress. It involves activating endogenous phytohormones (e.g., abscisic acid: ABA, gibberellins: GA, and auxin), and antioxidant enzymes (e.g., glutathione, peroxidase, and catalases) [15, 17, 18]. Alternatives such as exogenous plant growth regulators (PGRs) have recently been suggested to ameliorate the negative effects of flooding stress in plants. According to NASA, approximately 17 million km^2^ of the worldwide land area has been exposed to flooding [7, 19, 20]. However, among flooding conditions, waterlogging (WL) is a more common problem than submergence. WL stress in field conditions occurs for several reasons including overflow of rivers and heavy rainfall [9].

The physiological response of different crops to WL is variable. Soybean is one of the most important crops due to its high nutritional value [9]. In South Korea, soybean is not only regarded as an important field crop due to its high nutritional value, but it is also regarded as a higher income crop than paddy field crops such as rice [21]. According to a study by Nguyen et al. [5], soybean yield was 17% (vegetative stage) and 50% (reproductive stage) lower when exposed to WL stress conditions than when exposed to non-stress conditions. Soybean yield is also estimated to have decreased by 25% due to flooding stress in Asia, North America, and other regions of the world where soybean is rotated with rice in paddy fields [22]. Oosterhuis et al. [23] and Mustafa and Komatsu [22] reported reductions in soybean yield of 17–43% during the vegetative stage and 50–56% during the reproductive stage, respectively, due to flooding stress. Thus, several soybean breeders have been developed as tolerant varieties against WL stress and the identification of tolerance mechanisms under WL conditions has been studied [5, 9, 24, 25]. Our research team recently reported the physiological differences between a WL-tolerant soybean variety and a WL susceptible soybean variety and confirmed significant alteration in the different endogenous hormone levels (ABA, ET, GA, salicylic acid: SA, and jasmonic acid: JA) in contrasting soybean lines as well as the differences in antioxidant activities and root architecture such as lateral roots and aerenchyma cells [9]. Among several physiological responses, flooding responds to different ET levels. ET is biosynthesized by a short haul pathway in comparison to other plant hormones and is produced by 1-aminocyclopropane-1-carboxylic acid oxidation; therefore, oxygen is the main component for the production of ET [26, 27]. Plants may not produce enough ET without a supply of oxygen. However, ET production is significantly increased under WL conditions due to increased 1-aminocyclopropane-1-carboxylic acid synthase [26]. Furthermore, ET has been known to mitigate WL stress via the development of aerenchyma cells with crosstalk in oscillation with ABA, GA, indole-acetic-acid (IAA), and kinetin (KT) [15, 28, 29]. Conversely, ET can interact with JA and SA to induce the development of adventitious roots and aerenchyma cells in soybean [30, 31]. Therefore, the present study aimed to confirm the effect of exogenously applied PGRs (including ET) during WL stress by evaluating phenotypic characteristics. We then carried out a further experiment to identify the influence of morphological, physiological, and genetic responses of soybean plants through the exogenous application of selected PGRs among various plant hormones.

## Methods

### Selection of proper plant hormone

In the present study, we performed two experiments. Experiment I (EP I) was conducted to screen the appropriate PGRs to soybean plants to enhance resistance against WL stress conditions. Thus, we applied different types of PGRs such as ABA, ET (ethephon; ETP), GA_4_, IAA, KT, JA, and SA to soybean plants grown under WL stress conditions. Experiment II (EP II) was carried out to identify the physiological and biochemical mechanisms of phytohormone application during WL stress mitigation.

### Plant growth condition and application of PGRs (EP I)

We used the Daewon soybean variety (*Glycine max* L.) as the plant material because it is the most common variety of soybean and is broadly grown in South Korea. The seeds were donated by the National Institute of Crop Science, Rural Development Administration, South Korea. The seed surface was sterilized with 70% ethanol and then thoroughly rinsed with autoclaved double distilled water. Seeds were sown in plastic trays (50 holes, 40 cm × 20 cm), filled with autoclaved horticultural soil (Tobirang; Baekkwang Fertility, South Korea), and grown in a greenhouse located at the Kyungpook National University, Daegu, South Korea. Uniformly grown seedlings were transferred to plastic pots (six holes, 455 mm × 340 mm × 180 mm) 10 days after germination. When the soybean plant reached the V2 growth stage, we applied WL stress for 2 weeks (14 days). PGRs were applied to soybean seedlings 1 h after the WL treatment. Detailed information about PGRs applied is provided in Additional file 1: Table S1.

### Evaluation of resistance to WL stress (EP I)

To evaluate the mitigation effects on WL stress, we measured growth attributes, such as plant height, chlorophyll content, and chlorophyll fluorescence, during and after WL treatment. The chlorophyll content was measured with a chlorophyll content meter (CCM-300; Opti-Sciences, USA) and chlorophyll fluorescence data were recorded with a chlorophyll fluorimeter (OS5p+; Opti-Sciences, USA). The selection of the proper plant hormones to enhance the resistance of soybean to WL stress was conducted three times under greenhouse conditions and each experimental set consisted of three replications.

### Plant growth condition and PGRs application (EP II)

We confirmed the stress resistance effect during EP I. ETP application resulted in higher resistance to WL stress in the soybean than in the other PGRs treatments. Thus, we selected ETP and applied three different concentrations of ETP to soybean plants to identify the physiological and biochemical mechanisms against WL stress.

### Plant growth condition and ETP application (EP II)

We used the same seeds, soil, and pots in EP II as in EP I for seed germination; however, the seeds were grown in a growth chamber (Day 30 °C [14 h]/Night 22 °C [10 h], relative humidity 70%, light intensity 1000 μmol m^− 2^ s^− 1^) to collect accurate data. After the germination of seeds, uniformly growing soybean seedlings were selected and transplanted into the six-hole pots (the same size as in EP I) and maintained in the growth chamber. WL stress was applied to soybean plants during the V2 stage (fully developed trifoliate leaf at node immediately above the unifoliate node) and the water level was maintained at 10–15 cm above the soil surface for 10 days. Three different concentrations of ETP (50 μM, 100 μM, and 200 μM) were sprayed on the soybean shoot areas 1 h after subjection to WL (Additional file 1: Table S1).

### Analysis of chlorophyll content and chlorophyll fluorescence (EP II)

Chlorophyll content and fluorescence data were measured at 5, 10, and 15 days after WL. The chlorophyll content and fluorescence data were measured using the same methods as those mentioned for EP I. Data were collected three times and each replication was composed of seven plants (*n* = 7).

### Endogenous hormones analysis (EP II)

To analyze endogenous GA contents, we harvested shoot samples at 5, 10, and 15 days after the WL treatment, and harvested shoot samples grown under non-stress conditions at the same time periods. Plant samples were immediately placed in liquid nitrogen followed by freeze-drying (ISE Bondiro Freeze Dryer; Operon, South Korea) for 5 days. Thoroughly dried plant samples were ground into a fine powder, which was used for GA analysis. A 0.3 g dried sample was used for GA analysis and followed the same analysis protocol as that described by Kim et al. [9]. Endogenous GA content was analyzed by gas chromatography–mass spectroscopy with selective ion monitoring. In particular, endogenous GA_4_, GA_9,_ and GA_34_ contents were calculated from the peak area ratios of 284/286, 298/300, and 506/508, respectively. Data were collected three times (*n* = 3) and Duncan’s multiple range test (DMRT) was conducted for comparison among treatments. The condition of each instrument for hormone analysis is provided in Additional file 2: Table S2.

### Protein sample preparation (EP II)

Soybean leaf samples were washed twice with ice cold phosphate-buffered saline solution (in molecular cloning), sonicated for 10 s using a Sonoplus (Bandelin Electronic, Germany), and homogenized directly with a mortar-driven homogenizer (PowerGen125; Fisher Scientific, USA) in sample lysis solution composed of 7 M urea, 2 M thiourea containing 4% (*w*/*v*) 3-([3-cholamidopropy] dimethyammonio)-1-propanesulfonate, 1% (w/v) dithiothreitol, 2% (*v*/v) pharmalyte, and 1 mM benzamidine. Occasionally, a bead beater was used for lysis of rigid cells. Proteins were extracted for 1 h at 25 °C by vortexing. After centrifugation at 15,000×*g* for 1 h at 15 °C, the insoluble material was discarded, and the soluble fraction was used for two-dimensional gel electrophoresis. The protein concentration was assayed by the Bradford method [32].

### 2D PAGE (EP II)

Immobilized pH gradient dry strips (4–10 NL immobilized pH gradient, 24 cm, Genomine, Korea) were equilibrated for 12–16 h with 7 M urea, 2 M thiourea containing 2% 3-([3-cholamidopropy] dimethyammonio)-1-propanesulfonate, 1% dithiothreitol, and 1% pharmalyte, and loaded with 200 μg of sample. Isoelectric focusing was performed at 20 °C using a Multiphor II electrophoresis unit and EPS 3500 XL power supply (Amersham Biosciences, UK) following the manufacturer’s instructions. For isoelectric focusing, the voltage was linearly increased from 150 to 3500 V over 3 h for sample entry followed by a constant 3500 V, with focusing complete after 96 kV/h. Prior to the second dimension, strips were incubated for 10 min in equilibration buffer (50 mM Tris-Cl, pH 6.8 containing 6 M urea, 2% sodium dodecyl sulfate [SDS], and 30% glycerol), first with 1% dithiothreitol and second with 2.5% iodoacetamide. Equilibrated strips were inserted onto SDS-PAGE gels (20 × 24 cm, 10–16%). SDS-PAGE was performed using the Hoefer DALT 2D system (Amersham Biosciences, UK) following the manufacturer’s instruction. Two dimensional gels were run at 20 °C for 1700 V/h and then the 2D gels were stained with colloidal Coomassie brilliant blue as described by Oakley et al. [33], although the fixing and sensitization step with glutaraldehyde was omitted.

### Image analysis and identification of proteins (EP II)

Quantitative analysis of digitized images was performed using the PDQuest software program (version 7.0, Bio-Rad, USA) according to the protocols provided by the manufacturer. The quantity of each spot was normalized by total valid spot intensity. Protein spots were selected for the significant expression variation deviated over two-fold in its expression level compared to the control or normal sample.

For protein identification by peptide mass fingerprinting, protein spots were excised, digested with trypsin (Promega, Madison, WI), mixed with cyano-4-hydroxycinnamic acid in 50% acetonitrile/0.1% TFA, and subjected to matrix assisted laser desorption/ionization-time of flight analysis (Microflex LRF 20; Bruker Daltonics, USA) [34]. Spectra were collected from 300 shots per spectrum over m/z range 600–3000 and calibrated by two-point internal calibration using Trypsin auto-digestion peaks (m/z 842.5099, 2211.1046). The peak list was generated using Flex Analysis 3.0. The threshold used for peak-picking was as follows: 500 for minimum resolution of monoisotopic mass and 5 for S/N. The search program MASCOT, developed by Matrixscience (http://www.matrixscience.com/), was used for protein identification by peptide mass fingerprinting. The following parameters were used for the database search: trypsin as the cleaving enzyme, a maximum of one missed cleavage, iodoacetamide as a complete modification, oxidation as a partial modification, monoisotopic masses, and a mass tolerance of ±0.1 Da. The peptide mass fingerprinting acceptance criterion was probability scoring.

### Root phenotype (EP II)

We used the same soybean variety, but used a different soil type to measure root phenotypic difference among treatments (control and different concentrations of ETP). The sterilized soybean seeds were propagated in the six-hole pots (455 mm × 340 mm × 180 mm), which contained thoroughly washed and decomposed granite soils (overall size was 7–9 mm) to reduce root sample loss. To prevent the soil from drying out, enough water was supplied in the morning (0800–0900 h) and evening (1800–1900 h). When the soybean plant reached the V2 stage, the WL treatment was applied to each soybean plant for 15 days. During the WL period, the water level was maintained daily at 10–15 cm above the soil surface and the root samples were collected at 5-day intervals after the WL treatment until 15 days. The decomposed granite soil was carefully removed from the pots and the root samples washed twice with distilled water. Images of the clean root samples were captured with a digital camera (COOLPIX A; Nikon, Japan) at a mini studio (W 70 cm × L 100 cm). The image data was analyzed by Flower Shape Analysis System software (www.kazusa.or.jp/phenotyping/picasos/) to measure the root surface area (RSA) [35].

### Antioxidant activity and mRNA expression level (EP II)

To measure the stress response in soybean plants, we used the leaf samples. During the V2 stage, the WL treatment was applied to soybean plants for 2 days. We collected leaf samples at 1-day intervals and the same experiment was conducted three times. The fresh leaf and root samples were used to determine glutathione (GSH) and glutathione reductase (GR) activity. Briefly, 100 mg of fresh leaf samples were homogenized in buffer containing 50 mM Tris HCl (pH 7.0), 3 mM MgCl_2_, 1 mM EDTA, and 1.0% PVP. The homogenized samples were then centrifuged at 10,000 rpm for 15 min at 4 °C. We used the Bradford assay for quantification of total protein content [32]. The reduced GSH was estimated by following the protocol described by Ellman [36]. The homogenate was collected by grinding leaf samples with the addition of 3 mL of 5% (*v*/v) trichloroacetic acid. The supernatant (0.1 mL) was decanted into a tube containing 3 mL of 150 mM NaH_2_PO_4_ (pH 7.4). Subsequently, 500 μL of 5.5′-dithiobis (2-nitrobenzoic acid) (DTNB; 75.3 mg of DTNB dissolved in 30 mL of 100 mM of phosphate buffer, pH 6.8) was added to the suspension and it was incubated at 30 ± 2 °C for 5 min. The absorbance was measured at 412 nm and GSH contents were estimated by comparing with the standard curve. GR activity was measured by the protocol described by Garlberg and Mannervik [37]. A total of 150 μL of enzyme was reacted with 1 mL of reaction mixture containing 1 mM EDTA, 3 mM MgCl_2_, 0.5 mM oxidized glutathione, 0.1 M HEPES pH 7.8, and 0.2 mM NADPH. GR activity was measured by NADPH oxidation and monitored by decreased absorbance at 340 nm for 2 min. To investigate changes in gene mRNA expression among treatments, total RNA was isolated from fresh soybean leaf tissue using TRIzol (Invitrogen, USA). Briefly, fresh soybean leaf tissue was finely ground using liquid nitrogen and 1 mL of TRIzol was added immediately. Samples were then centrifuged for 5 min at 13,000 rpm and 4 °C. The supernatant was transferred to a new 1.7 ml tube and chloroform and isopropanol were added for phase separation and RNA precipitation, respectively. Centrifuge steps were carried out in between. Isolated RNA pellets were washed with 75% DEPC EtOH, dissolved in RNase-free water, and treated with DNaseI. Total RNA was used for cDNA synthesis following the manufacturer’s protocol (cDNA synthesis kit, Phile, Korea). The cDNA was used as a template for real-time PCR (Eco™ Real-Time PCR, Illumina, USA). During the real-time PCR process, 2× Quantispeed SYBR Mix (PhileKoea) was used as the reaction mixture, and the PCR was conducted according to the manufacturer’s protocol. GmUBI was used as the reference gene for data normalization and all data were replicated three times. Detailed primer information for the real-time PCR process is listed in Additional file 3: Table S3.

### Statistical analysis

The experiments (EP I and EP II) were conducted three times with three replications under greenhouse and growth chamber conditions, respectively. The experiments of antioxidant activity and determination of mRNA expression level, and SNO related gene expression levels were conducted two times with three replications under a greenhouse condition. Analysis of variance (ANOVA) was tested at *P* < 0.05 to evaluate significant difference among treatments, periods, replications, and treatments by periods. Comparison among treatments was conducted by the DMRT and the SAS 9.1 software program was used for statistical analysis.

## Results

### Plant growth characteristics with and without waterlogging (WL) stress (EP I)

**Fig. 1:**
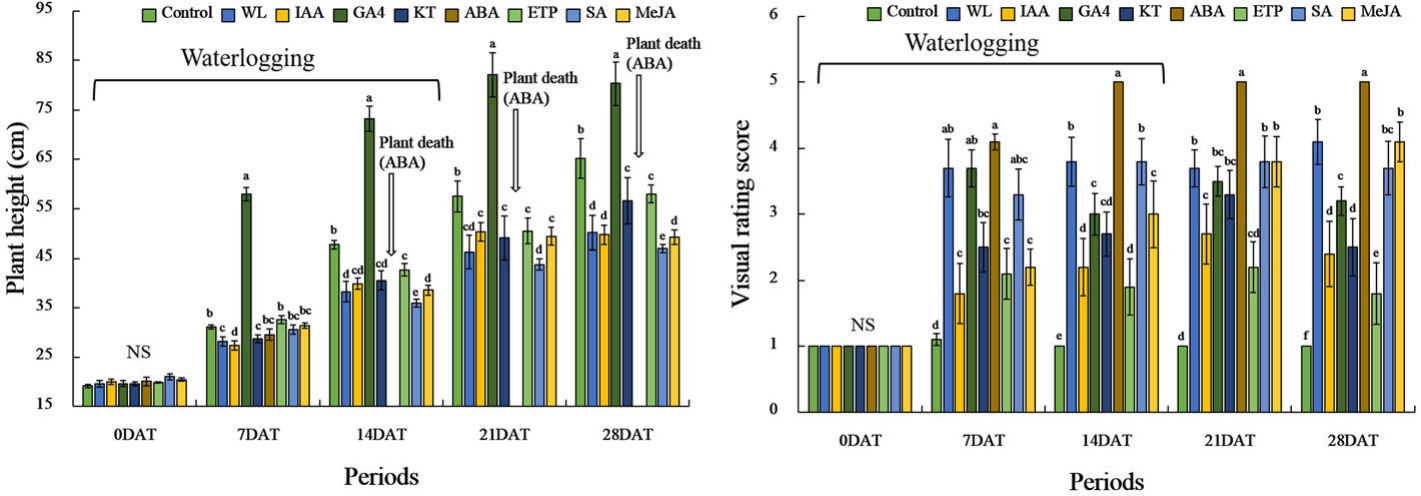
Soybean plant height and visual rating score during and after waterlogging (WL) treatment. WL treatments were maintained for 14 days. In the figure, NS indicated no significant difference among treatments and plant death means no plant survival at ABA treatment. Visual rating scores: 1, no plant damage (plants healthy); 2, initial signs of wilting and curling; 3, most leaves are wilting and drooping; 4, all leaves have wilted and many are brown and crispy; and 5, death of growing point. Data were collected three times and are presented as the average ± standard error (*n* = 10)

**Fig. 2:**
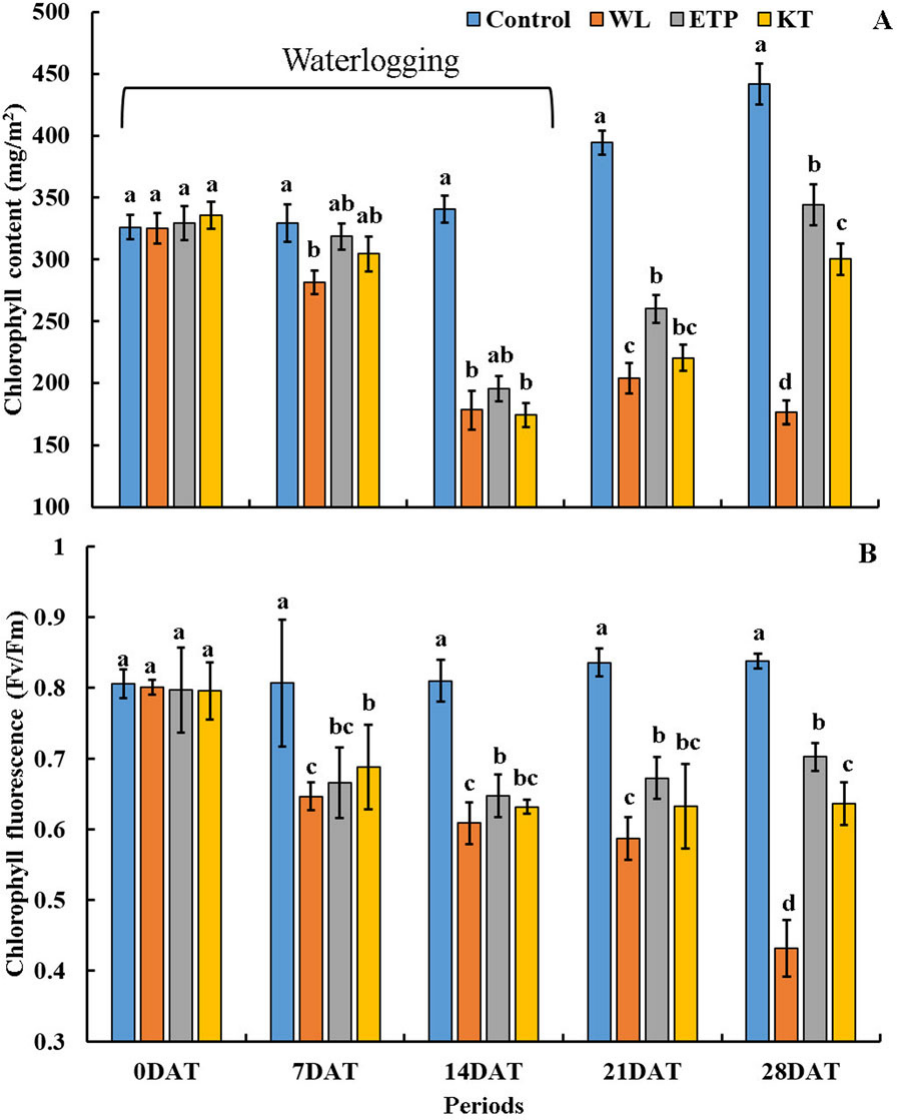
Changes in chlorophyll contents and chlorophyll fluorescence during and after WL treatment. Each capital letters, **a** and **b** indicated chlorophyll content and chlorophyll fluorescence, respectively. WL treatments were maintained for 14 days during both data collections. WL indicates the WL only treatment. Different letters above error bars indicate significant differences at *P* < 0.05 and data were analyzed by Duncan’s multiple range test. Data were collected three times and are presented as the average ± standard error (*n* = 10)

To evaluate the effects of the PGRs, we monitored the plant height with and without WL stress. When we applied PGRs to soybean plant (0 DAT), the plant height was not significantly different among PGR treatments. However, the plant height was significantly (*P* < 0.05) increased by 48% (28 DAT, minimum value)–56% (7 DAT, maximum value) in the GA_4_ treatment at 7, 14, 21, and 28 days after treatment (DAT) (Fig. 1), with the plants not showing WL resistance (data not shown). A higher plant height was observed in ETP and KT applications than that seen in the WL only treatment, whereas IAA, SA, and Methyl-JA applied to soybean plants did not cause any differences or decreases in plant height compared to that in the only WL treatment (Fig. 1; 28 DAT). Soybean plants died after 14 days of WL treatment when 100 μM ABA treatment was applied (Fig. 1). Results of the visual rating score (VRS) showed that WL-treated plants presented around 3.5–4.0 VRS, whereas the control plants showed 1.0 VRS at all time points. Among PGR-treated plants, IAA, KT, GA, and ETP-treated plants showed improved VRSs compared to that of WL (Fig. 1). In particular, ETP-treated plants showed the lowest VRS compared to IAA, KT, and GA applications (Fig. 1). Based on the results of Fig. 1 (plant height and VRS), we decided on IAA and ETP as provisional candidate materials; thus, we carried out additional experiments using KT and ETP. The chlorophyll content and chlorophyll fluorescence after ETP and KT applications were measured, with no significant difference shown before the WL treatment (Fig. 2). The chlorophyll content significantly decreased in all WL-treated plants. However, ETP and KT-treated plants had higher chlorophyll contents than that of the WL only treatment during the experimental periods (Fig. 2a). Moreover, the chlorophyll content was higher in the ETP application than in the KT application (Fig. 2a). Moreover, chlorophyll fluorescence of ETP and KT applied to soybean plants showed increased levels (45–50% increase at 28 DAT) than that of the WL only treatment. In particular, chlorophyll fluorescence in ETP-treated plants was higher than that in those treated with KT (Fig. 2b). Overall, the ETP treatment showed a promising result in extending WL stress tolerance in soybeans compared to other PGRs; therefore, ETP was selected for further experiments.

### Effect of ETP application on plant growth characteristics with and without WL stress (EP II)

**Fig. 3:**
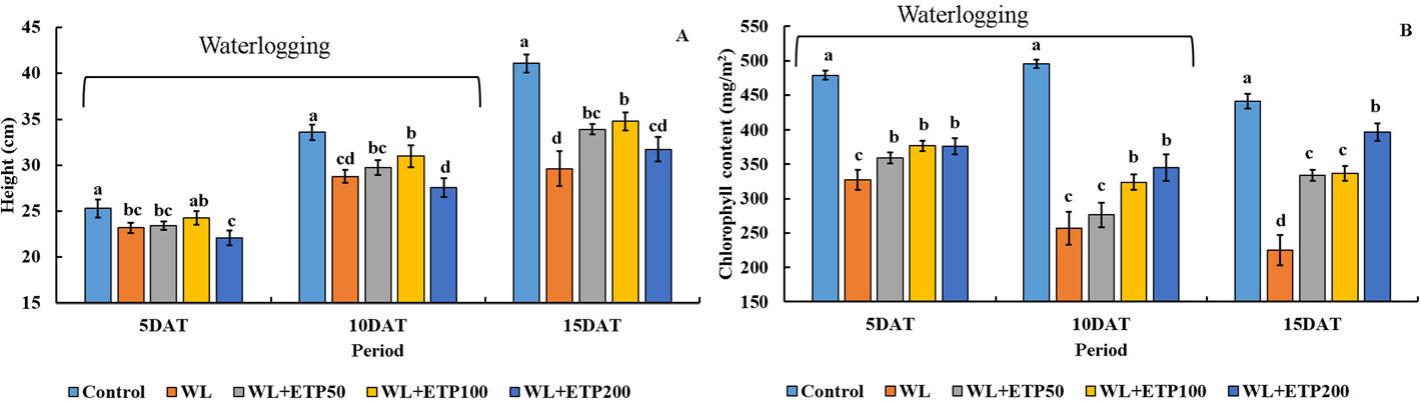
Influence of various concentrations of ethephon treatment on plant height (**a**) and chlorophyll content (**b**). Soybean plants were exposed to WL for 10 days. Data were collected in 5-day intervals from three replicate samples and are shown as the average ± standard error (*n* = 10). In the figures, different letters indicate significant difference at *P* < 0.05 and data were analyzed by Duncan’s multiple range test (DMRT). WL = waterlogging; DAT = days after treatment; and ETP = ethephon

Through the EPI, we confirmed the effects of ETP application to soybean plants under waterlogging conditions via several phenotypic variables, such as plant height, visual rating score, chlorophyll content, and chlorophyll fluorescence. These results were similar to our previous report [9]; thus, we departmentalized the concentration of ETP to identify an appropriate concentration and waterlogging tolerance mechanism. Three different concentrations of ETP (50 μM [ETP50], 100 μM [ETP100], and 200 μM [ETP200]) was applied to soybean plants. The plant height was lower (7.2–23.7%) than that of the control under both stress conditions (WL only or WL with ETP treatments) (Fig. 3a). When we compared the plant height between WL only and WL with ETP applications, it was higher in ETP50 and ETP100 applications compared to that in the only WL application. On the other hand, a decreased plant height was observed in the WL with ETP200 application compared to that in the WL only treatment at 5 DAT and 10 DAT (Fig. 3a). Higher chlorophyll contents (10.3–54.7%) were observed under non-stress conditions during all time periods (Fig. 3b). The plants that received ETP had gradually higher chlorophyll content levels depending on the concentrations of ETP than those of the WL only treatment (Fig. 3b).

### Effect of ETP application on chlorophyll contents and fluorescence (EP II)

**Fig. 4:**
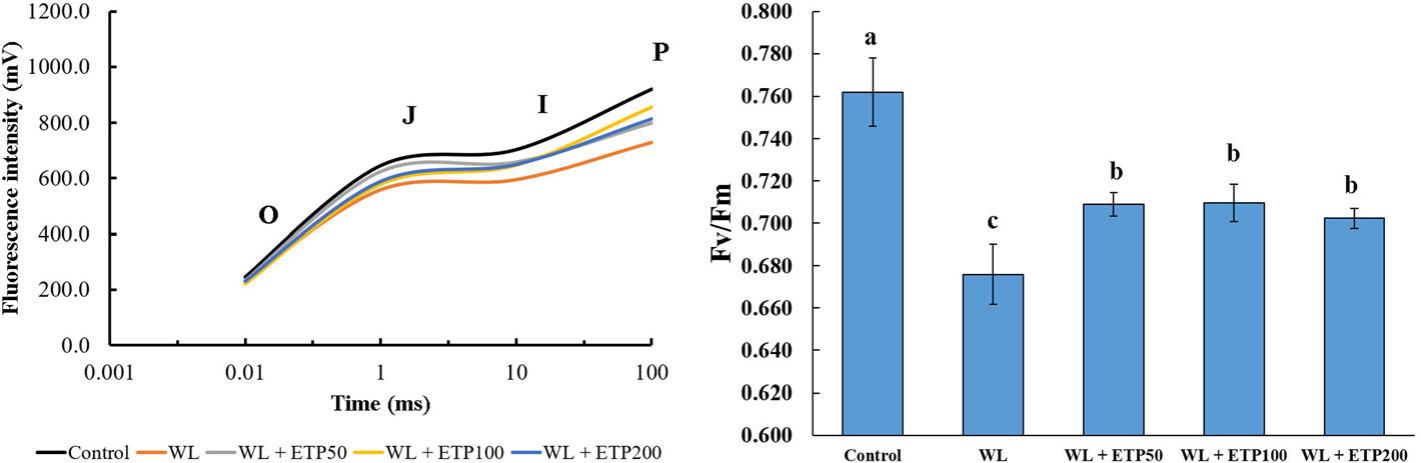
OJIP parameters and photosynthetic efficiency (Fv/Fm) in soybean plants at 15 days after WL. We measured OJIP data after 20 min of dark acclimation. Data were detected by three times and are presented as the average ± standard error (*n* = 10). WL = waterlogging; DAT = days after treatment; and ETP = ethephon

To evaluate the photosynthetic efficiency, we measured the chlorophyll fluorescence (Fv/Fm) at 15 days after WL treatment. Overall, lower OJIP curves were observed in the WL-treated and WL with ETP-treated plants than in the control (Fig. 4a). However, ETP-treated plants showed a slightly improved OIJP curve compared to that of WL-treated plants. In particular, with the phase from J to P showing differences between ETP-treated plants and WL-treated plants (Fig. 4a). Fv/Fm showed similar results to those of the OJIP. Non-stressed soybean plants showed higher levels of Fv/Fm (0.76) than the other treatments. Comparison among WL and WL + ETP treatments showed improved Fv/Fm in ETP-treated plants (Fig. 4b).

### Influence of ETP application on endogenous plant hormones (EP II)

**Fig. 5:**
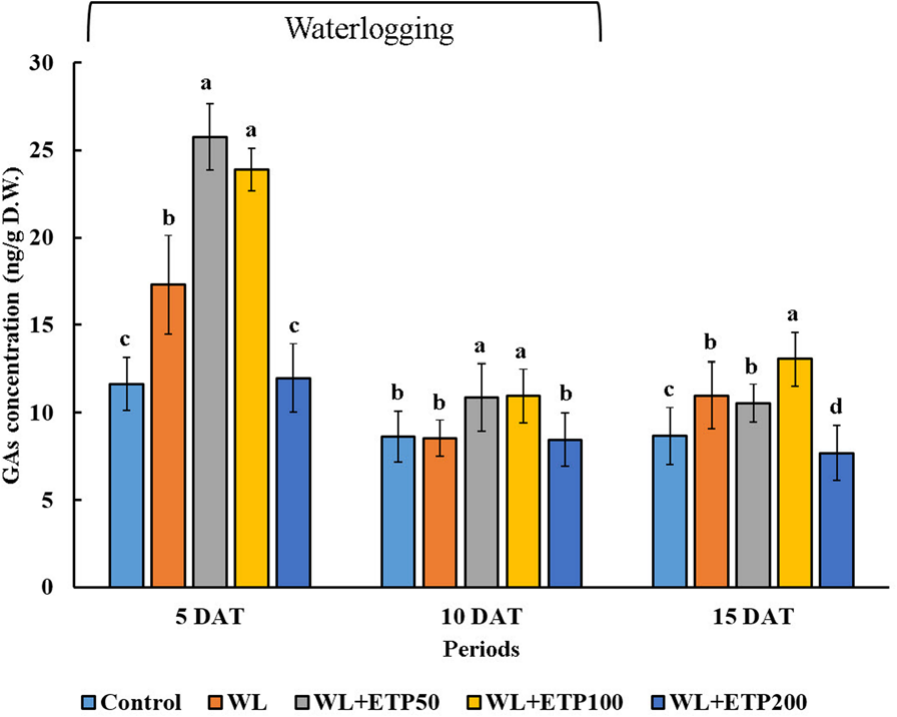
Influence of different concentrations of ethephon treatments on endogenous GAs levels. Soybean plants were exposed to WL for 15 days. Data were collected at 5-day intervals from three replicate samples. Different letters indicate significant differences at *P* < 0.05 and data were analyzed by Duncan’s multiple range test. WL = waterlogging; DAT = days after treatment; ETP = ethephon; and GAs concentration = sum of GA_4_, GA_9_, and GA_34_

We analyzed the levels of endogenous GA to elucidate physiological response during stress periods. In higher plants, endogenous bioactive GAs (GA_1_, GA_3_, GA_4,_ and GA_7_) are synthesized by two different pathways, one is the early 13-hydroxylation pathway and the other is the non-13-hydroxylation pathway [38]. According to a previous study [9], soybean mainly produces bioactive GA_4_ via the non-13-hydroxylation pathway, and thus we focused on the determination of bioactive GA_4_ and its intermediate precursor (GA_9_) and catabolite (GA_34_). In Fig. 5, the GAs indicate the sum of GA_4_, GA_9_, and GA_34_. The GA contents showed relatively lower levels in the control and ETP200 applications than in the other treatments (WL only, ETP50, and ETP100), whereas the GA contents were significantly higher in the ETP50 and ETP100 treatments than in the WL only treatment at 5 DAT. Enhanced levels of GA were observed in ETP50 and ETP100 at 10DAT (Fig. 5).

### Change in amino acid contents (EP II)

**Fig. 6:**
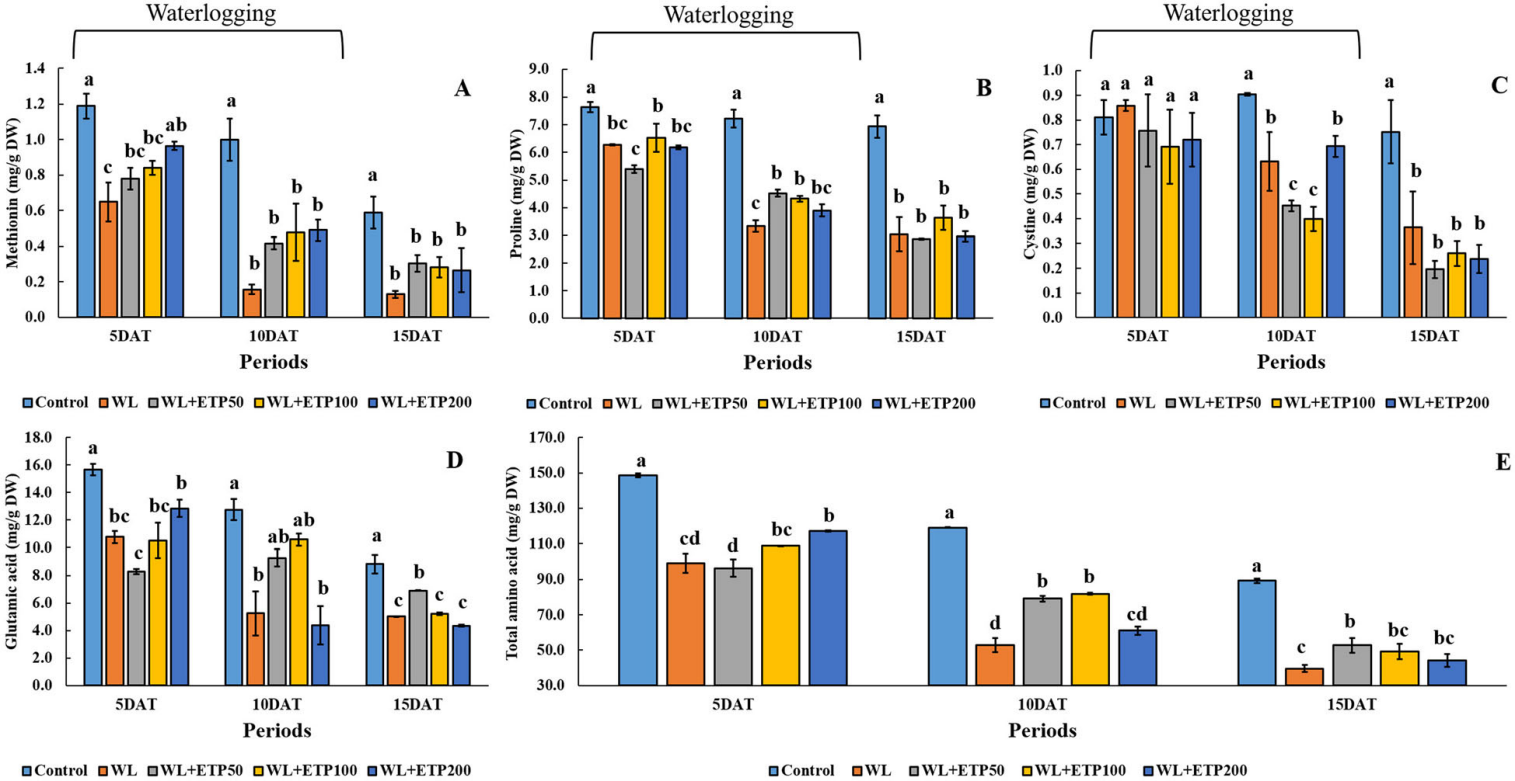
Amino acid contents in soybean plants during and after WL treatment. Data was collected at 5-day intervals for up to 15 days and the standard error was collected from two replicates. In the figure, each capital letters indicated amino acid content (**A**: methionin, **B**: proline, **C**: cystine, **D**: glutamic acid, and **E**: sum of the 16 amino acid contents). WL = waterlogging; DAT = days after treatment; and ETP = ethephon. Total amino acid = sum of 16 amino acids (Ala, Arg, Asp, Cys, Glu, Gly, Ile, Leu, Lys, Met, Phe, Pro, Ser, Thr, Tyr, and Val)

Methionine, proline, cysteine, and glutamic acid are known as abiotic stress responses. The methionine content was significantly decreased in soybean plants grown under the WL only treatment. However, higher methionine content was measured in all ETP-treated plants than that in soybean plants grown under the WL only treatment (Fig. 6a). The same pattern was observed at all time points (5 DAT, 10 DAT, and 15 DAT). Proline and glutamic acid contents were significantly lower (18.4–53.6%, *P < 0.05*) in the only WL and WL with ETP application groups than in the control, and the same tendency was observed at all time points (Fig. 6b, d). In ETP-treated plants, proline and glutamic acid contents showed statistically similar or slightly higher results than in the WL only treatment (Fig. 6b, d). Cysteine content did not show any differences among treatments in 5 DAT. When comparing the WL only and WL with ETP treatments, cysteine contents were significantly lower at 10 DAT and no differences among treatments were found at 15 DAT (Fig. 6c). The sum of the 16 amino acids (total amino acids) contents showed consistent results (Fig. 6e). Total amino acid contents were significantly lower in WL-treated plants than that of the control plants, whereas concentrations of amino acids were significantly higher in the ETP-treated plants than in the WL only treatment plants at 10 DAT and 15 DAT (Fig. 6e).

### Root surface area (RSA) (EP II)

**Fig. 7:**
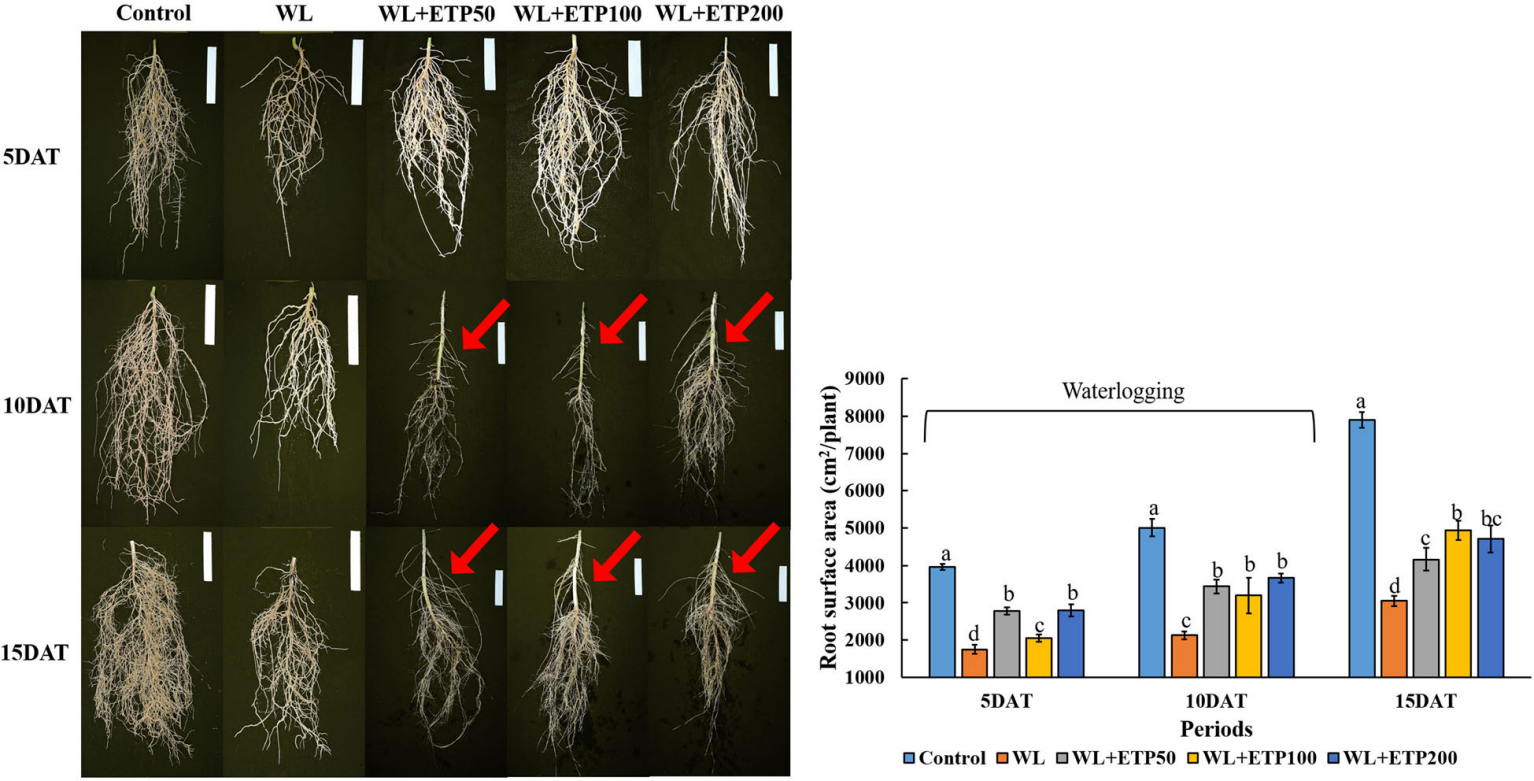
Root image and root surface area (RSA) in soybean plants during and after WL treatment. White bar signifies 5 cm^2^ (W: 1 cm × L: 5 cm). Vertical bar with error bar shows the average with standard error (*n* = 5). In the figure, red arrows indicated adventitious roots. During the same stress exposure period, different letters indicate significant difference at *P* < 0.05 and data were analyzed by Duncan’s multiple range test. WL = waterlogging; DAT = days after treatment; and ETP = ethephon

Adventitious roots were not observed in the control soybean plants, whereas well developed adventitious roots were observed in the WL with ETP-treated plants compared to those in the WL-treated plants. The same results were observed at all time periods (Fig. 7). RSA analysis revealed that the control soybean plants had significantly higher RSAs than the WL- and the WL with ETP-treated plants at 5 DAT, 10 DAT, and 15 DAT. The RSA was higher in the WL with ETP-treated plants than in the WL-treated plants (Fig. 7). In particular, the application of ETP100 and ETP200 resulted in significantly higher RSAs than that did that of ETP50 at 15 DAT (Fig. 7).

### Proteomics expression during WL treatments (EP II)

**Fig. 8:**
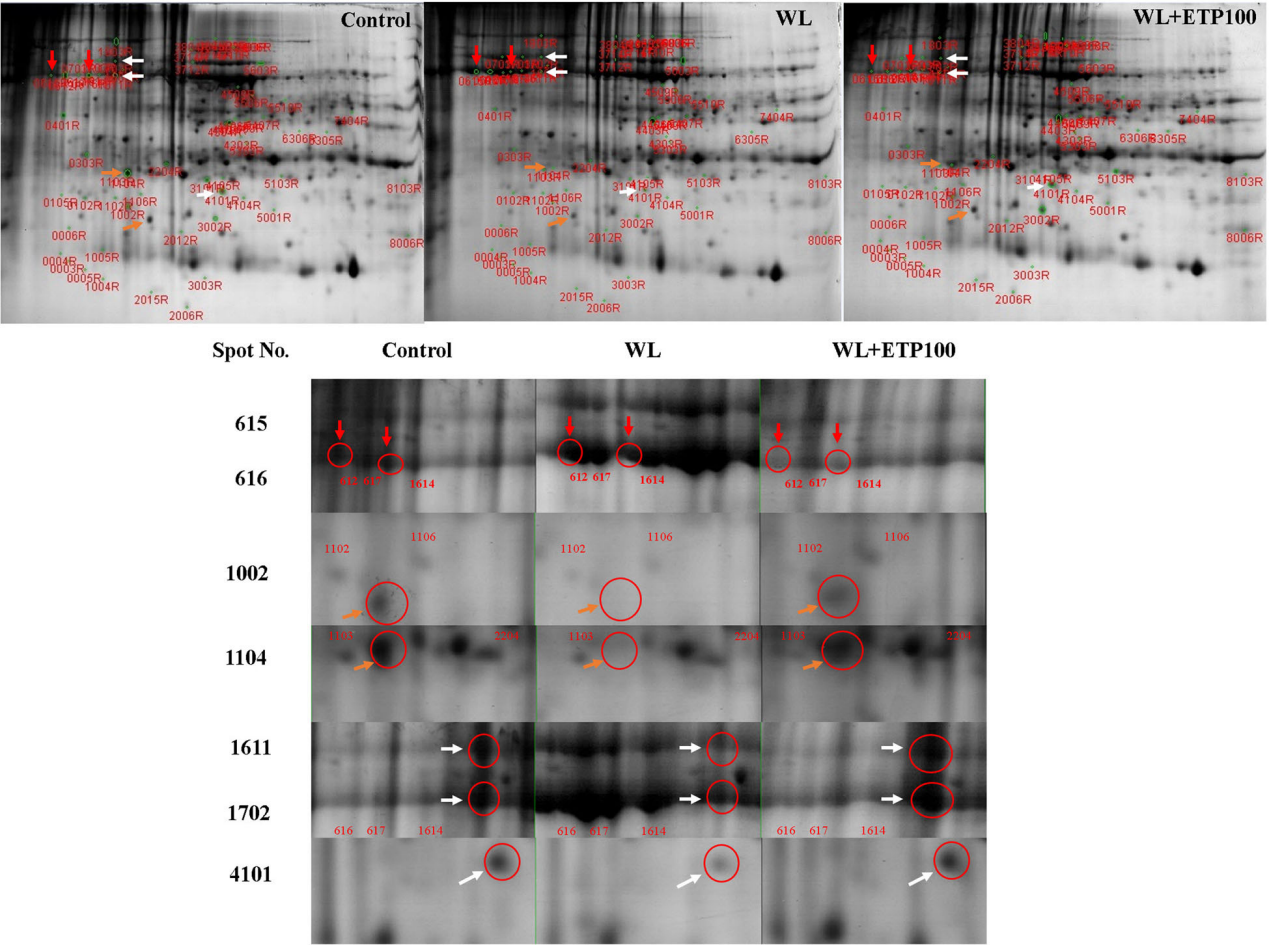
Protein expression levels in soybean plants. Orange and white colored arrows indicate down-regulated proteins in the WL only treatment whereas up-regulated proteins were observed in WL with ethephon-treated plants. Red colored arrows show highly up-regulated proteins in the WL only treatment

To identify the protein expression pattern under WL treatments, soybean plants were exposed to WL for 10 days. Two-dimensional gel electrophoresis images showed that 63 different proteins expression patterns were measured (Fig. 8). Among these 63 proteins, we selected seven interesting spots for investigation. The seven spots were identified as ribulose-1, 5-bisphosphate carboxylase/oxygenase large subunit (Spot No. 615, 616, 1611, and 1702), trypsin inhibitor A (Spot No. 1002), glutathione S-transferase DHAR2 (Spot No. 1104), and glycoprotein (Spot No. 4101) (Table 1). Among the seven identified proteins, two proteins (Spot No. 615 and 616; red arrows) were up-regulated in the WL only treatment compared to the control and the WL with ETP treatment, whereas five proteins (Spot No. 1002, 1104, 1611, 1702, and 4101; orange and white arrows) were down-regulated in the WL only treatment compared to the control and WL with ETP treatment (Fig. 8). Moreover, expressions of these proteins were recovered in the ETP100 treatment. Therefore, our data suggests that these proteins would be participating in inducing the resistance to WL stress.

**Table 1:** Protein information of soybean plant exposed to waterlogging stress for 10 days

Spot No.	MW	PI	Protein name	Score	Accession No.
615	53.03	6.04	ribulose-1,5-bisphosphate carboxylase/oxygenase large subunit, partial (chloroplast)	225	YP_538747
616	51.57	6.04	ribulose-1,5-bisphosphate carboxylase/oxygenase large subunit, partial (chloroplast)	212	SBO07506
1002	17.01	5.03	trypsin inhibitor A	119	XP_003532237
1104	20.77	5.03	glutathione s-transferase DHAR2	116	AJE59632
1611	54.88	4.94	ribulose-1,5-bisphosphate carboxylase/oxygenase large subunit, partial (chloroplast)	204	CAB08877
170	66.80	4.94	ribulose-1,5-bisphosphate carboxylase/oxygenase large subunit	216	CAB08877
4101	18.55	5.91	glycoprotein	148	NP_001241536

Accession number (GI number), *MW* molecular weight, *PI* isoeletronic point

### Antioxidant activity and mRNA expression level (EP II)

**Fig. 9:**
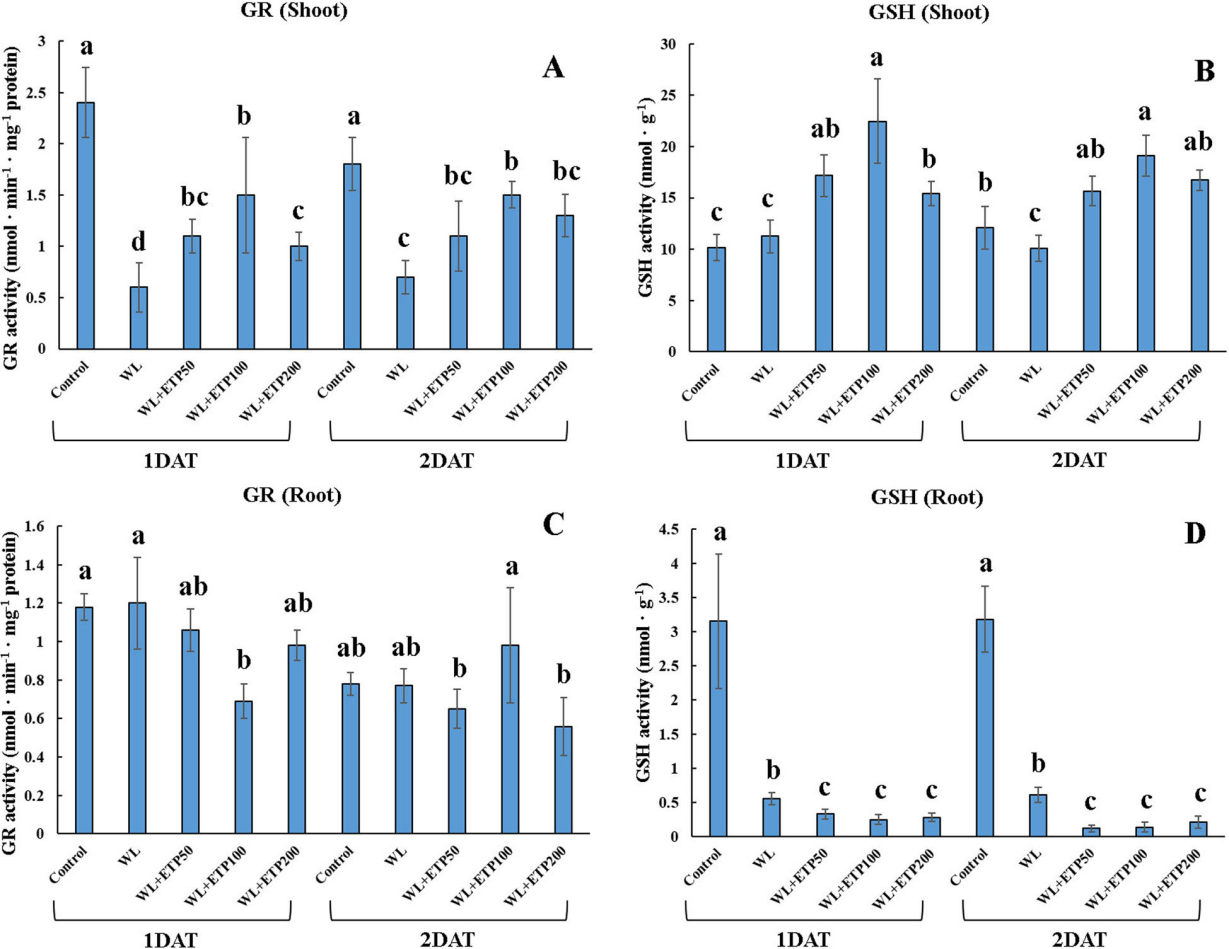
Influence of various concentrations of ethephon treatments on antioxidants (GR and GSH) activity. Soybean plants were exposed to WL for 2 days. Data were collected at 1-day intervals from three replicates. In the figure, capital letter **A** and **B** meant GR and GSH activity in the shoot area and capital letter **C** and **D** meant GR and GSH activity in the root area. In each figure, different letters indicate significant difference at *P <* 0.05 and data were analyzed by Duncan’s multiple range test. WL = waterlogging; DAT = days after treatment; and ETP = ethephon

**Fig. 10:**
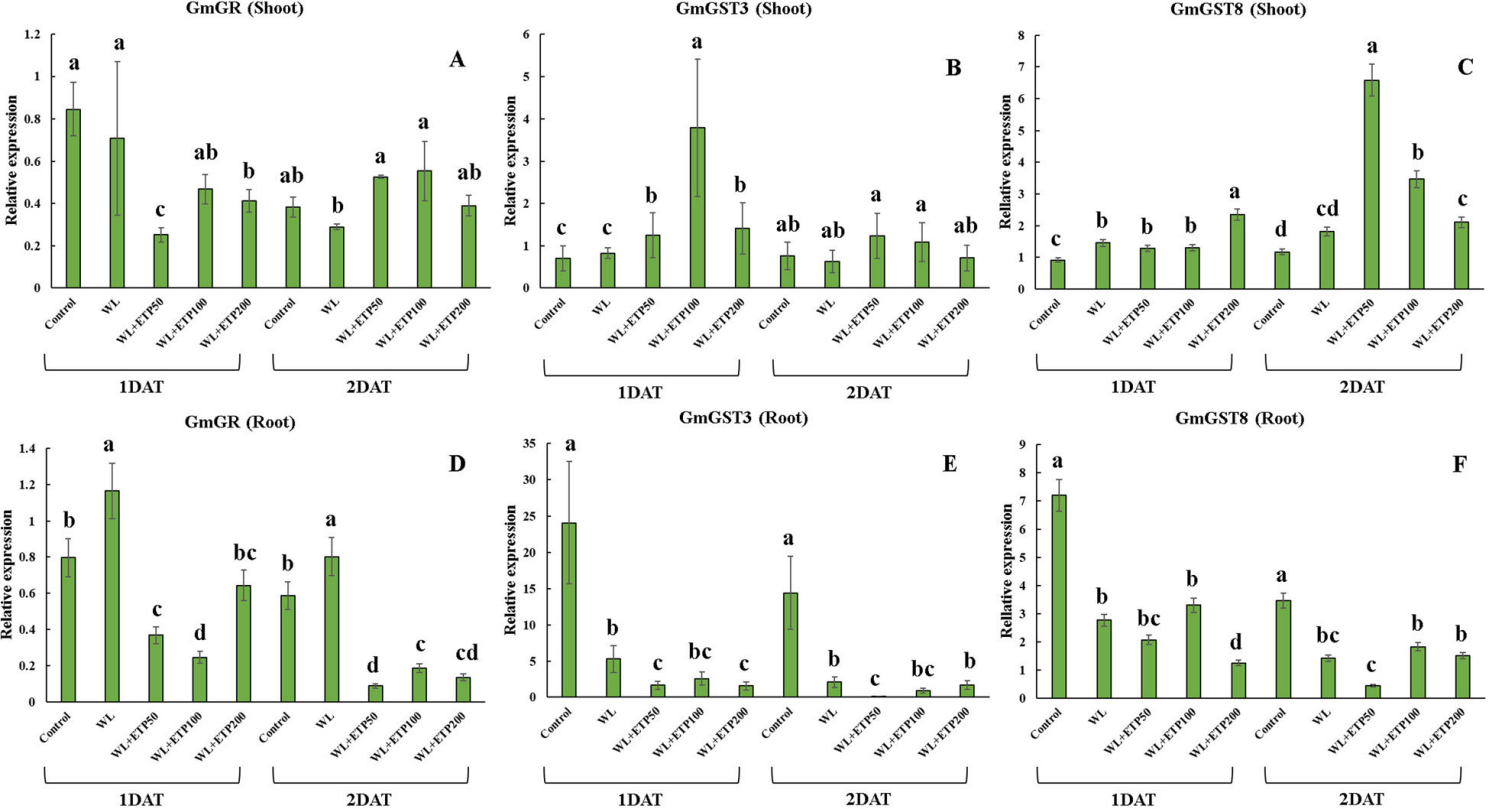
Influence of various concentrations of ethephon treatments on specific gene expression levels (*GmGR*, *GmGST3*, and *GmGST8*). Soybean plants were exposed to WL for 2 days. Data were collected at 1-day intervals from three replicates. In the figure, capital letter **A**, **B** and **C** meant the gene expression level in the shoot area and capital letter **D**, **E** and **F** meant the gene expression level in the root area. Different letters indicate significant difference at *P* < 0.05 and data were analyzed by Duncan’s multiple range test. WL = waterlogging; DAT = days after treatment; and ETP = ethephon

According to our 2-DE results, the glutathione S-transferase DHAR2 protein was down-regulated in the WL-treated plants, but was recovered by ETP application. Thus, we measured the GSH and GR activities at the genetic and enzymatic levels. The GR activity of soybean shoots was lower in the WL- and WL with ETP-treated plants than that of the control plants (Fig. 9a). However, GR activity was higher in the WL with ETP-treated than that in the WL-treated plants (Fig. 9a). The GR activity in the shoots revealed a similar pattern between 1 DAT and 2 DAT, whereas the GR activity in the roots did not show a regular pattern (Fig. 9c). Expression levels of *GmGR* showed differences between the shoots and roots. In the shoots, the expression levels of *GmGR* were lower in the WL with ETP-treated plants than in the control at 1 DAT (Fig. 10a). The expression levels of *GmGR* were lower in the WL with ETP-treated plants than in the WL-treated plants, whereas the expression levels of *GmGR* were dramatically changed at 2 DAT. The most increased expression levels were observed in the ETP50 or ETP100 WL treatments (Fig. 10a). In the roots, the expression levels of *GmGR* were higher in the WL-treated plants than the other treatments, whereas the WL with ETP-treated plants showed lower expression levels than the control and WL-treated plants (Fig. 10d).

GSH activity showed significant difference (*P <* 0.05) between the shoots and roots. In the shoots, GSH activity was significantly higher in the WL with ETP-treated plants than that of the control and WL-treated plants, with a similar pattern observed during both stress exposure periods (Fig. 9b). However, GSH activity was significantly lower (*P <* 0.05) in the WL with ETP-treated plants than in the control and WL-treated plants, with the same pattern repeated during both exposure periods (Fig. 9d). At the genetic level, GSH activity was very well described in *GmGST3* compared to in *GmGST8* for both the shoots and roots (Fig. 10b, c, e, and f). Similarly, for GSH activity, the WL with ETP-treated plants (shoots) showed higher (*P* < 0.05) expression levels of *GmGST3*, while lower expression levels (*P* < 0.05) of *GmGST3* were measured in WL with ETP-treated plants (roots) (Fig. 10b, e). In 1 DAT, expression levels of *GmGST8* in shoots were higher in the WL only and WL with ETP treatments than in the control. However, no significant differences were found among the WL only, WL with ETP50, and WL with ETP100 treatments (Fig. 10c). Similarly, the WL with ETP-treated plants showed different expression patterns between the shoots and roots. At 2 DAT, higher expression levels of *GmGST8* were detected in WL with ETP-treated plants than were detected in the control and WL only treatment (Fig. 10c). In particular, WL with ETP50-treated plants showed the highest expression levels (Fig. 10c). In contrast, in the roots, the expression level of *GmGST8* showed a similar tendency to GSH activity in soybean root. The WL with ETP-treated plants showed significantly lower expression levels than did the control (Fig. 10f). However, the expression levels of *GmGST8* in the roots did not show a constant tendency among WL with ETP-treated plants (Fig. 10f).

## Discussion

The mechanism behind tolerance to flooding is very well known at the genetic level in rice plants, thus several genes (*Sub1A*, *SNORKEL1* [*SK1*], and *SNORKEL2* [*SK2*]) related to flooding stress in rice plants were identified by QTL mapping [28, 42]. However, the flooding mechanism in soybean plants is not yet fully understood. Previously, Nguyen et al. [5] conducted quantitative trait locus (QTL) mapping using contrasting soybean varieties against WL to identify the flooding mechanism in soybean plants. To date, several QTLs involved in WL resistance [5, 39, 40] have been reported. However, the QTLs were not narrowed down enough to identify the physiological mechanism involved or for use in marker-assisted selection [9].

In a previous study, significantly increased ET production and GA_4_ content, as well as decreased ABA, were observed in a WL-tolerant soybean variety. Based on previous research, we evaluated the effect of exogenous treatment with PGRs on soybean plants under WL stress in the present study. According to our results (plant height, VRS, chlorophyll content, and chlorophyll fluorescence), GA, KT, and ETP application showed a higher resistance against WL than that of the control (Figs. 1 and 2). Among the three PGR applications, we selected ETP as the candidate because, GA was not suitable to use in the agricultural industry due to large internode growth [41]. The plants that received ETP showed greater shoot growth as well as an observed decrease in leaf chlorosis than did KT-treated plants. Moreover, ETP application was theoretically closer to the results of our previous study [9]. Thus, we selected ETP as the final candidate from among several PGR treatments originally tested.

The overall plant height was higher in ETP with WL-treated plants than in the WL-treated plants. Therefore, we needed to provide evidence related to the tolerance mechanism in wetland plants to prove this difference between ETP with WL and WL only treatments. In rice plants, different resistance strategies have been identified depending on water level [28]. Hattori et al. [42] reported that increased shoot length was measured in WL-tolerant rice varieties under submergence conditions and physiological responses were induced by *SK1* and *SK2* genes. The *SK1* and *SK2* genes are included in ET response factors (ERF). Thus, this gene accumulates GA or induces GA signal transduction to increase internode elongation. Finally, different shoot lengths have been measured in deep-water rice plants [43]. We previously reported similar results [9]. According to Kim et al. [9], significantly higher bioactive GA_4_ was observed in WL-tolerant soybean variety than in the WL susceptible soybean variety. Therefore, based on the above evidence, we assumed that the increased shoot length from ETP application would be related to the stress avoidance strategy in soybean plants, and that endogenous bioactive GAs would participate in this response.

Photosynthesis in plants is driven mainly by two different photosynthetic apparatus: photosystem II (PSII) and photosystem (PSI) in the thylakoid membrane [44–46]. The main role of PSII is for the oxidation of water to oxygen and protons, followed by transferring protons to ATP synthase for generating ATP [45]. OJIP parameters indicate photosynthesis efficiency and have been broadly used for anticipating plant stress [47]. In the present study, when soybean plants were exposed to WL stress for 15 days, the OJIP parameter was lower in WL only and WL with ETP treatments than that of the control; however, the ETP-treated plants showed less damage than the WL-treated plants (Fig. 4). A similar pattern was measured in chlorophyll fluorescence. The Fv/Fm values showed a decreasing tendency in the WL- and WL with ETP-treated plants; however, ETP-treated plants showed a relatively higher value than did the WL treatment (Fig. 4). If plants are exposed to salt stress, plants have decreased photosynthetic activity due to the limitation of photosynthetic electron transport [48]. In particular, oxygen evolution activity and PSII electron transport activity are significantly decreased; thus, significantly decreased fluorescence yields at J, I, and P are observed [48]. Our results show that ETP application participates in WL stress mitigation by reinforcing the photosynthetic pigment or enhancing the electron transport, because ETP-treated plants showed relatively less reduction in photosynthesis related parameters.

The plant hormone GA is known as a key signaling molecule that is involved in various physiological responses, such as seed germination, cell division, cell elongation, and stress responses [9, 38]. In particular, GA is heavily involved in water stress escape strategies in rice plants [28]. The up-regulation of various GAs have been reported in rice plants during submergence and assist rice plants in exposing some of its parts to the atmosphere via hyper elongation of the stem [28, 42]. Finally, this physiological and morphological response offers resistance against submergence conditions in rice plants [49, 50]. Likewise, increased bioactive GA_4_ contents were detected in a WL-tolerant soybean cultivar (PI408105A) [9]; therefore, these authors hypothesized that increased bioactive GA content was one of the main responses to WL stress in soybean. In the present study, significantly increased GA contents were detected in WL with ETP50- and ETP100-treated plants (Fig. 5). In particular, GA concentrations were higher in the relatively short-term (5 DAT) stress exposure. Soybean plants produce adventitious roots to survive WL stress, which is a very common response under flooding stress [5, 9, 41, 51], and these reactions were accompanied by stem swelling and root penetration, thereby leading to the development of adventitious roots [51]. Ethylene is a key regulator of adventitious root formation. Based on a study by Steffens et al. [52], penetration or growth of adventitious roots were significantly increased in the presence of GA_3_ and ETP-treated rice plants. Similar results were reported in other crops including petunias, tomatoes, and mung beans [53–56]. Based on previously documented evidence and our results, we hypothesized that exogenously applied ET participated in the accumulation of endogenous GA, and thus increased GA promotion not only increased plant height but also the formation of adventitious roots. Therefore, this physiological phenomenon is one response to escape WL stress in soybeans.

WL condition in soybean plant is a restricting factor for root growth. When soybean plants were exposed to the WL treatment for 5 days, decreased root size was observed in the WL only treatment; however, the ETP-treated plants showed greater RSA than did WL-treated plants (Fig. 7). RSA also decreased in the WL only treatment whereas ETP-treated plants showed increased RSA in all time periods. Under WL conditions, soil pores are covered with water; therefore, plants receive limited oxygen uptake and gas exchange [13, 52]. Thus, plants produce adventitious roots to combat against stress condition. ET and GA regulate formation, number, and length of adventitious roots synergistically. Therefore, exogenous ET source application stimulates the accumulation in GA levels in soybean plants, which improve RSA. Thus, we assumed that improved RSA will participate possibility of more oxygen uptake thus plant showed resistance. As we determined via root images was decreased in the WL only treatment. However, it was improved by ETP application. Thus, our results suggested that soybean root growth was inhibited under the WL treatment. However, ETP treatment induced improved root growth therefore, ETP application to soybean plants induced resistance against WL stress conditions.

Total amino acid contents were decreased by WL; however, they were higher in ETP treatments than that in WL only treatments. In particular, methionine content revealed significant difference between WL only and WL with ETP treatments. ETP-treated plants showed higher methionine content that that of non-ETP-treated plants during WL conditions. According to a study by Koppitz [57], oxygen deficiency of underground organs due to flooding creates changes in amino acid contents and carbohydrate metabolism because oxygen participates in more than 200 different reactions, such as mitochondria respiration, oxidation, and oxygenation. Hence, high contents of amino acids have been observed in the common reed (*Phragmites australis*) under flooding conditions with decreased carbohydrates contents measured [57]. These results were quite different to the data obtained from the present study. According to our results, total amino acid contents and specific amino acid (methionine, proline, cysteine, and glutamic) contents decreased under both WL treatments. This distinction among amino acid contents between common reed and soybean were caused by different resistance against hypoxia or anoxia. Common reed grows in sunny, wetland habitats; fresh water marshes; and riverbanks; thus, it is well adapted to soil hypoxia [58]. Hypoxia-tolerant species can produce sufficient amounts of ATP via anaerobic fermentation whereas large amounts of carbohydrates are required to meet demands for metabolic energy [57]. Therefore, although common reed grows under flooding conditions, it produces large quantities of amino acids and huge consumption of carbohydrates. Conversely, the soybean plant is regarded as a hypoxia-sensitive species; thus, significantly decreased amino acid contents were observed. Overall amino acid contents were decreased in both WL treatments; however, ETP-treated soybean plant showed less decreases than did WL-treated plants. According to a study by Kim et al. [9], WL-tolerant soybean plants showed significantly higher endogenous ET production than did WL-susceptible soybean plants whereas significantly lower methionine contents were observed in WL-tolerant soybean plants than in WL-susceptible soybean plants. Based on previous studies, we assumed that WL-tolerant soybean plants accumulated more endogenous ET to resist WL condition. Therefore, a lower methionine content was observed in WL-tolerant plants because methionine is a precursor of ET. We induced high concentrations of endogenous ET production artificially in soybean plants resistant to WL treatment. After the ET donor source, ETP, was applied to soybean plants after WL, we found changes in amino acid contents and protein expression in soybean plants. According to our results, two proteins were down-regulated in ETP applied soybean plants compared to the control and WL-treated plants, which was identified as RuBisCO protein. The other two proteins (Spot no. 1611 and 1702) were up-regulated in ETP applied soybean plants compared to WL-treated plants, which was also known as RuBisCO protein. RuBisCO initiates carbon assimilation via carboxylation of RuBP (ribulose-1, 5-bisphophate) during C_3_ photosynthesis [59]. RuBisCO is located in the chloroplasts of plants and not only participates in carbon fixation during photosynthesis but also regulates the release of used CO_2_, NH_3,_ and energy during photorespiration [59–61]. Normally, RuBisCO is the largest protein in C_3_ or C_4_ plant leaves; thus, approximately 30–50% of proteins are known as RuBisCO [62]. For this reason, the expression of RuBisCO protein showed high frequency in 2-DE analysis [59, 62]. In a study by Krishnan and Natarajan [63], a TCA/acetone extraction procedure with a phytic acid treatment was used to deplete RuBisCO to increase the accuracy of soybean leaf protein. Therefore, we regarded that our compatible results, up- or down-regulation of RuBisCO protein in WL with ETP treatment, were not the major findings to represent the overall results. Thus, we focused more on other proteins. Three proteins, trypsin inhibitor A, glutathione S-transferase DHAR2, and glycoprotein, were up-regulated in ETP-treated plants compared to WL-treated plants (Fig. 8). Among these, in particular, we focused on the glutathione S-transferase DHAR2 because this is known as a scavenger of ROS and reactive nitrogen species via ascorbate-glutathione cycle during hydrogen peroxide [64–66]. According to a study by Foyer and Noctor [21], oxidative stress has several hallmarks, such as increased oxidative load, oxidative damage to cellular components, and accumulation of damaged cellular components. This can lead to loss of function in plant cells, and, ultimately, plants face death. Ascorbate and glutathione interdependently catalase in high capacity redox homeostatic H_2_O_2_; thus, these two enzymes are closely linked to each other [64, 67, 68]. The main function of glutathione S-transferase is regeneration of ascorbate; therefore, DHAR is one of several routes for GSH oxidation [69]. GSH dependent enzymes, glutathione S-transferases (GSTs), are included in plants for detoxification; thus, GST-encoding genes are strongly induced by oxidative stress [67, 70, 71]. GSTs are abundant proteins and are involved in xenobiotics detoxification, as well as act as antioxidants by combining with oxidative degradation productions, acting as a glutathione peroxidase, and removing lipid peroxides [67, 71]. For these reasons, we focused on the analysis of gene expressions involved in GSH activity (*GmGR* and *GmGSTs*) to elucidate genetic differences due to ETP supplementation after WL treatment. The expression level of *GmGST3* was up-regulated in all ETP-treated plants (shoots) compared to the control and WL only treatment, and increased GSH activity was observed in ETP-treated plants (shoots). On the other hands, reduced GSH activity, as well as decreased expression levels of *GmGST3*, was observed in soybean plants, especially in the roots. According to Herschbach et al. [72], GSH is involved in the cell proliferation of meristematic root cells and synthesizes in the roots as well as being transported from the shoot via the phloem. Therefore, we assumed that different expression patterns of *GmGST3* between the shoot and root were caused by different ROS contents. Namely, the ROS content might be increased in the shoot area under WL condition, so soybean plant need to concentrate on ROS scavengers in the shoot area, and therefore GSH transport to the root area should be reduced. ETP-treated soybean plants showed increased GSH activity and expression level of GmGST3; thus, ETP-treated soybean plants showed obvious differences between the shoots and roots. Thus, higher expression levels of *GmGST3* were observed in the shoot area than in the root area. This result indicated that ETP application to soybean plants after WL could stimulate the up-regulation of GST3 expression. In other words, if soybean plants are subjected to WL conditions, ROS is rapidly accumulated in the plant cell. Therefore, the plant has to properly remove ROS to survive unfavorable environmental conditions; thus, plants operate defense mechanisms through the production of antioxidants such as CAT, APX, and GSH. In WL conditions, ethylene application induces stress mitigation in soybean plants. The application of artificial ethylene, ETP, induces the up-regulation of GST3 and GST8 in soybean plants; thus, ETP-treated soybean plants showed increased protein levels (glutathione S-transferase DHAR2). Therefore, for soybean plants under WL conditions, genetic (GST3 and GST8) and proteomic (glutathione S-transferase DHAR2) changes due to ETP application derive various phenotypic differences such as well-developed adventitious roots, increased amino acid content, and enhanced chlorophyll fluorescence reaction.

## Conclusion

Based on our results, we can summarize that under WL treatment, soybean plants experienced oxidative stress due to limited mitochondrial respiration; therefore, increased ROS decreased or suppressed morphological or physiological phenomena, such as plant height, RSA, chlorophyll content, chlorophyll fluorescence, and amino acid contents. However, ETP-treated plants showed alterations to their morphological and physiological parameters compared to WL-treated plants because ETP application induced a higher activity of antioxidants, such as GR and GSH, compared to that in the WL-treated plants. Moreover, for the antioxidant activity (GR and GSH), ETP application could stimulate a higher expression of GST3 and GST8 in the shoot area; thus, increased GST3 and GST8 consecutively induced 1) increased GSH activity (shoots), 2) decreased ROS, 3) mitigation of cell damage in photosynthetic apparatus, and 4) improved phenotype. Therefore, we hypothesize that exogenous ET application to soybean plants growing under WL stress triggers beneficial effects against WL via improved ROS scavenging, especially the up-regulation of GSH genes.

## Additional files

Additional file 1: **Table S1.** Information on plant growth regulators (PGRs) and application concentrations. (DOCX 18 kb)

Additional file 2: **Table S2.** Gas chromatography–mass spectroscopy with selective ion monitoring and HPLC conditions for endogenous GA analysis. (DOCX 17 kb)

Additional file 3: **Table S3.** Primer sequences for qRT-PCR. (DOCX 18 kb)

## Abbreviations

ABA: Abscisic acid
EP I: Experiment I
EP II: Experiment II
ET: Ethylene
ETP: Ethephon
GA: Gibberellins
GR: Glutathione reductase
GSH: Glutathione
GSTs: Glutathione S-transferases
IAA: Indole-acetic acid
JA: Jasmonic acid
KT: Kinetin
PGRs: Plant growth regulators
ROS: Reactive oxygen species
RSA: Root surface area
SA: Salicylic acid
*SK1*: *SNORKEL1*
*SK2*: *SNORKEL2*
WL: Waterlogging
