# Supplementary material for: Exo-ethylene application mitigates waterlogging stress in soybean (Glycine max L.)
Source: BMC Plant Biol. 2018 Oct 22;18:254. doi: 10.1186/s12870-018-1457-4 (PMC6198449; doi:10.1186/s12870-018-1457-4)
Supplement: Supplementary file 1 — Table S1. Information on plant growth regulators (PGRs) and application concentrations. (DOCX 18 kb) [file 12870_2018_1457_MOESM1_ESM.docx]

Additional file 1: **Table S1.** Information of plant growth regulators (PGRs) and its application concentration.

| **Concentration** | **PGRs (group)** |
| --- | --- |
| 100 µM | Indole-3-acetic acid (IAA 98%, Sigma Aldrich, USA), (Auxin) |
|  | Gibberellin A4 (GA_4_ > 90%, Sigma Aldrich, USA), (Gibberellin) |
|  | Kinetin (KT 99%, Sigma Aldrich, USA), (Cytokinins) |
|  | Abscisic acid (ABA 98%, Sigma Aldrich, USA), (Abscisic acid) |
|  | Ethephon (ETP 39%, Kyung Nong, South Korea), (Ethylene) |
|  | Salicylic acid (SA 99%, Sigma Aldrich, USA), (Salicylic acid) |
|  | Methyl Jasmonic acid (MJA 95%, Sigma Aldrich, USA), (Jasmonic acid) |

100 µM PGRs was used for selection of aproprite PGR for experiment I (EPI) after that three different concentration of ETP (50 µM, 100 µM and 200 µM) were used during experiment (EPII).
