# Supplementary material for: Exo-ethylene application mitigates waterlogging stress in soybean (Glycine max L.)
Source: BMC Plant Biol. 2018 Oct 22;18:254. doi: 10.1186/s12870-018-1457-4 (PMC6198449; doi:10.1186/s12870-018-1457-4)
Supplement: Supplementary file 2 — Table S2. Gas chromatography–mass spectroscopy with selective ion monitoring and HPLC conditions for endogenous GA analysis. (DOCX 17 kb) [file 12870_2018_1457_MOESM2_ESM.docx]

Additional file 2: **Table S2.** GC-MS-SIM and HPLC conditions for endogenous GA analysis.

| **GC-MS-SIM** | |
| --- | --- |
| **Model** | Hewlett-Packard 6890, 5973N Mass Selective Detector |
| **Column** | DB-1 capillary column (10 m x 0.25 mm, i.d. 0.25 μm film thickness) |
| **Carrier gas** | He (40 mL·$\min^{-1}$) |
| **Source tem.** | 250℃ |
| **Oven condition** | GA: 60℃ (1 min) → 15℃·min^-1^ → 200℃ (1 min) → 5℃·min^-1^ → 285℃ (5 min) |
| **Injector tem.** | 200℃ |
| **Ionizing voltage** | 70ev |
