# Supplementary material for: Exo-ethylene application mitigates waterlogging stress in soybean (Glycine max L.)
Source: BMC Plant Biol. 2018 Oct 22;18:254. doi: 10.1186/s12870-018-1457-4 (PMC6198449; doi:10.1186/s12870-018-1457-4)
Supplement: Supplementary file 3 — Table S3. Primer sequences for qRT-PCR. (DOCX 18 kb) [file 12870_2018_1457_MOESM3_ESM.docx]

Additional file 3: **Table S3**. Primer sequence for qRT-PCR

| **Accession No.** | **Name** | **Forward** | **Reverse** |
| --- | --- | --- | --- |
| LOC547906 | GmUBI | TCCAGAAGGAGTCAACTCTC | TGGATGTTCTTAGTCAGCGAG |
| X68819 | GmGST3 | ATGGCTTGGGTCAAGAGATG | CCAACCCACACAACCATAG |
| AF243363 | GmGST8 | AGGAGATTGGGTTGGTAGAT | TGAAAAAGGAGAAAACAGGA |
| L11632 | GmGR | CTCATTAAGGTCGTGTGTCAT | CAAATTCGATGCAGAGATTA |
